# Innovations in natural dye production: bridging tradition and modern technology

**DOI:** 10.3389/fpls.2025.1568094

**Published:** 2025-08-15

**Authors:** Shailendra Yadav, Harish Chandra Prajapati, Sankatha P. Sonkar, Rama Shankar Nigam, Rishikesh Chandravanshi, Chitrasen Gupta

**Affiliations:** ^1^ Department of Chemistry, Faculty of Basic Science, AKS University, Satna, MP, India; ^2^ Centre for Green Chemistry & Sustainability, AKS University, Satna, MP, India; ^3^ Department of Chemistry, Government P.G. College, Chunar, Mirzapur, UP, India; ^4^ Department of Chemistry, Kutir P.G. College, Jaunpur, UP, India

**Keywords:** natural dyes, sustainability, modern technology, enzymatic extraction, environment

## Abstract

Natural dyes have a rich historical significance, rooted in traditional practices that utilize plant materials, minerals, and organic substances to produce vibrant pigments. However, their use declined with the advent of synthetic dyes in the 19th century due to challenges in scalability, cost, and color consistency. Recent advancements in sustainability and modern technology have reignited interest in natural dyes, offering innovative solutions to overcome historical limitations. This review highlights traditional and contemporary extraction techniques, including solid-phase micro extraction, supercritical fluid extraction, pressurized-liquid extraction, and microwave-assisted extraction. Additionally, cutting-edge approaches such as grinding-assisted microwave irradiation and enzymatic extraction methods are examined for their ability to enhance yield, efficiency, and environmental sustainability. These modern techniques enable the utilization of unconventional sources, including agricultural waste and invasive species, thereby promoting sustainable dye

## 1 Introduction

Natural dyes possess a rich historical significance, deeply intertwined with cultural heritage and sustainable practices. For centuries, people in different communities around the world used natural resources to make dyes. They used plants, minerals, and other organic materials to create bright and long-lasting colors for textiles, artwork, and cosmetics. Synthetic dyes were introduced in the 19th century, which reduced the use of natural dyes. This happened because natural dyes were harder to produce on a large scale, more expensive, and less consistent in color. Natural colorants are obtained from natural sources through various extraction methods following the drying and grinding of plant materials. The selection of an appropriate solvent is based on the specific dye of interest, and extraction is performed using both conventional and advanced techniques (Zhang et al., 1994; Luque de Castro and Garcia-Ayuso, 1998; Sasidharan et al., 2011). The desired compounds, utilized for dyeing applications, are isolated using chromatographic or alternative separation methods. These isolated compounds are subsequently characterized through spectral analysis techniques (Yadav et al., 2023) recently, the heightened focus on sustainability, environmental responsibility, and ethical production has revitalized interest in natural dyes. Newer technologies are now enabling the evolution of this traditional practice into a progressive and sustainable industry (Gala et al., 2018). Present paper suggests a new advanced method grinding assisted microwave extraction of natural dye from biological materials.

## 2 Bridging tradition in natural dye production and applications

The shift from traditional to modern methods of natural dye production addresses several key limitations of traditional techniques, such as inconsistent color fastness, limited scalability, and environmental concerns. Traditional methods often rely on lengthy extraction processes, large amounts of water, and toxic mordants. Modern advancements, such as enzymatic extraction, microbial fermentation, and nanotechnology, enhance dye yield, improve color stability, and reduce environmental impact. For example, enzymatic extraction allows for more efficient pigment retrieval without hazardous chemicals, while microbial fermentation enables the sustainable production of bio-based dyes. Additionally, nano-enhanced dyeing techniques improve adherence to fabrics, reducing water and energy consumption. These innovations not only preserve the cultural heritage of natural dyeing but also make the process more viable for large-scale, eco-friendly textile production (Yusuf et al., 2016; Gala et al., 2018; Yadav et al., 2023).

Natural dyes are used in textiles, cosmetics, food, and sustainable packaging. Ethical fashion brands and the beauty industry increasingly favor plant-based pigments (Ghosh et al., 2022). Scientific breakthroughs, including microbial dye production and AI-assisted dye optimization, enhance the scalability and consistency of natural dyes. One report express that applied machine learning models to optimize parameters such as pH, temperature, and dyeing time, significantly improving color uniformity and reducing water consumption in plant-based textile dyeing (Zhou et al., 2023; Schweitzer et al. 2024).

Many cultures, including Indian block printers, Japanese indigo dyers, and African resist-dye artisans, have preserved and revived dyeing traditions. Governments and NGOs support artisans through training and global market access. However, challenges such as color inconsistency, limited scalability, and durability concerns remain. These problem can be solved by hybrid dyeing techniques, biodegradable textiles, and regenerative agriculture for dye plant cultivation (Alawa et al., 2013).

By blending tradition with innovation, natural dyeing continues to evolve as a viable alternative to synthetic dyes, promoting cultural heritage while advancing sustainability in various industries.

## 3 Natural dye extraction

**Figure 1:**
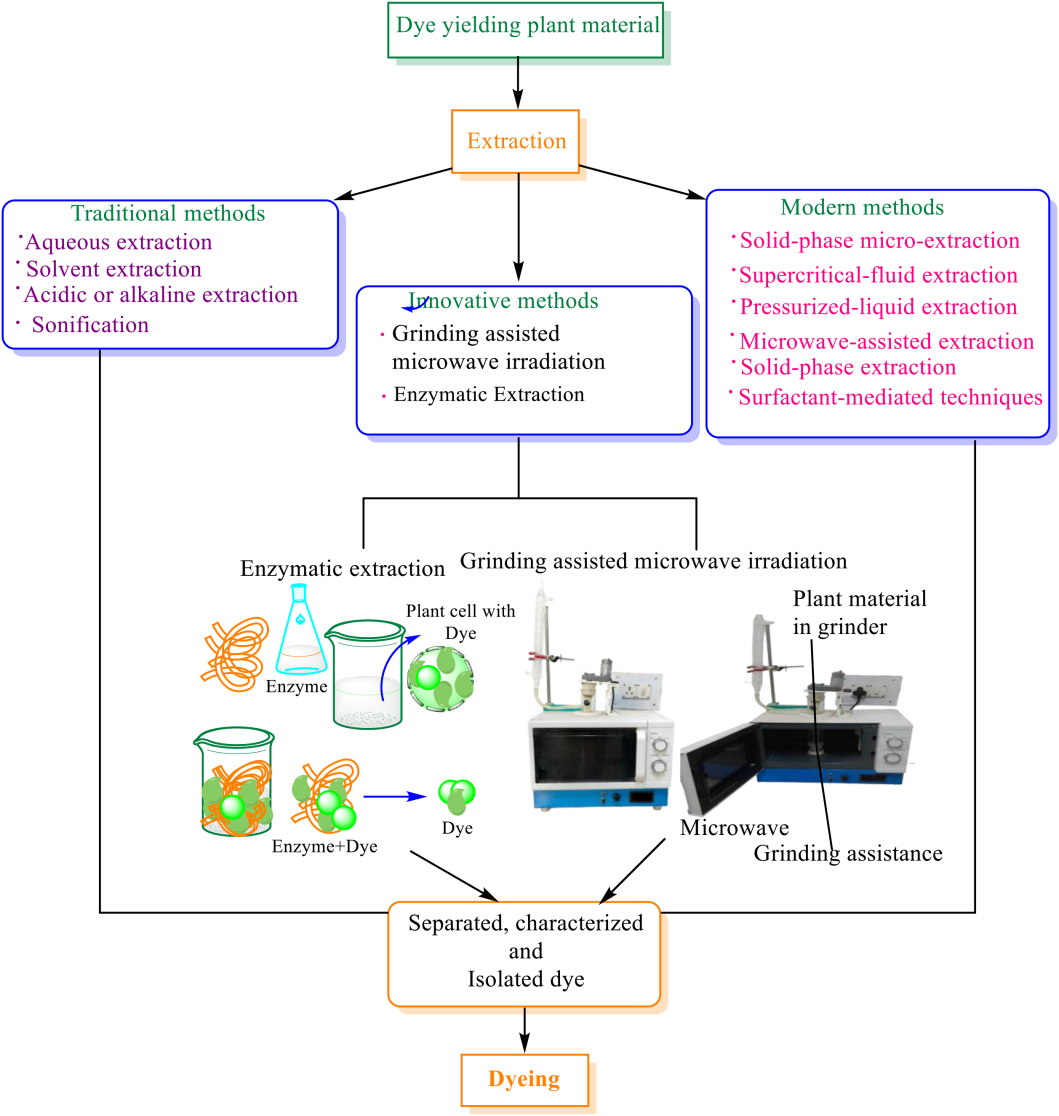
Traditional, modern and advanced methods of natural dye extraction.

Traditional methods of dye extraction, such as boiling or fermenting plant materials, often posed several challenges (Bart and Pilz, 2011; Pandey et al., 2020). These included inefficiencies, significant resource consumption, and inconsistent dye quality due to variations in processing conditions (Pranta and Rahaman, 2024). However, recent technologies have addressed many of these limitations, leading to more efficient, sustainable, and reliable processes **Figure 1** (Křížová, 2015; Affat, 2021; Hagan and Poulin, 2021; Slama et al., 2021).

### 3.1 Modern method

Contemporary extraction techniques surpass traditional methods due to their ability to achieve higher yields with reduced costs. Several studies have quantitatively compared modern dye extraction techniques based on yield, efficiency, and product quality. It was reported that microwave-assisted extraction (MAE) of Coleus atropurpureus yielded 42% more pigment compared to traditional boiling, with a reduction in extraction time by 60% (Watharkar et al., 2022). Similarly, enzymatic extraction methods using cellulase or pectinase have shown dye yield increases of 20–30% with enhanced color fastness up to 4.5–5 rating on the gray scale, compared to 3.0–3.5 for conventional methods (Rani et al., 2023). Grinding-assisted microwave extraction method have demonstrated 10-15% higher extraction efficiency and significantly lower solvent usage than stand-alone microwave or mechanical methods in early stage of study. Moreover, supercritical fluid extraction (SFE) using CO_2_ has been shown to recover 95–98% of target anthocyanins with minimal thermal degradation (Herrero et al., 2006) Key modern extraction methods are summarized below.

#### 3.1.1 Solid-phase micro extraction

SPME is a solvent-free extraction technique that integrates sampling, extraction, concentration, and sample introduction into a single step. The method relies on (a) the partitioning of the target analytes between the extraction phase and the sample matrix, and (b) the desorption of the concentrated analytes into the instrument’s storage or analytical system. This technique is simple, efficient, and enables the enrichment of analytes while minimizing solvent consumption. However, a limitation of SPME is the limited availability of commercially produced stationary phases (Kataoka et al., 2020).

#### 3.1.2 Supercritical fluid extraction

This technique leverages the significant solubility of target dyes in supercritical fluids, such as carbon dioxide (CO_2_), propane, butane, or ethylene. The dye interacts with the supercritical fluid within an extraction vessel under high pressure. SFE is an advanced separation method that exploits the enhanced solvating capacity of gases in their supercritical state, which occurs above their critical temperature and pressure. Carbon dioxide is the most widely used supercritical fluid due to its low critical temperature, non-flammability, low toxicity, and cost-effective availability in pure form. Typically, CO_2_ is employed within a temperature range of 40°C to 80°C and a pressure range of 35–75 MPa. A key advantage of this technique is that small variations in temperature and pressure result in significant changes in the density and solvating power of the supercritical fluid, enabling efficient extraction. However, SFE is not cost-effective in some applications, although the affordability of CO_2_ makes it a viable option for many practical uses (Vankar et al., 2001).

#### 3.1.3 Pressurized-liquid extraction

Pressurized-liquid extraction is a technique in which extraction is carried out under elevated temperatures and moderate to high pressure to enhance the efficiency of dye component recovery. This method is time-efficient and requires minimal solvent usage. The process begins by dispersing the sample with inert materials, such as sand, and placing the extraction material into a specialized vessel. An appropriate solvent is introduced during the static phase, which typically lasts between 0.5 and 21 minutes, while the system is heated to the desired temperature. Following this, the dynamic extraction phase begins, during which the solvent flows continuously through the material. The efficiency of this method depends on several factors, including the choice of solvent, temperature, pressure, and the nature of the adsorbents used. Key advantages of PLE include rapid extraction and reduced solvent consumption. However, the primary drawback of this technique is the need for expensive equipment and additional cleanup requirements (Osorio-Tobón et al., 2013)

#### 3.1.4 Microwave-assisted extraction

Microwave-assisted extraction is a technique that employs microwave radiation at varying power levels (e.g., 264 W, 400 W, and 600 W) in the presence of solvents to extract dye compounds from plant materials. In this process, microwave radiation generates heat within the extraction material and solvent, facilitating the transfer of target compounds into the solvent. This method is rapid and suitable for thermally unstable compounds. The efficiency of MAE depends on several factors, including the choice of solvent, extraction material, target compound properties, extraction time, temperature, and microwave power. MAE is employed in two configurations: closed-vessel and open-vessel systems.

The closed-vessel system offers advantages such as reduced solvent requirements and minimal loss of volatile compounds. However, it is limited in processing sample quantities. In contrast, the open-vessel system operates safely at atmospheric pressure, allowing reagents to be added during extraction. While the open system is limited to processing fewer samples simultaneously, the closed system is capable of handling multiple samples in parallel (Routray and Orsat, 2012; Osorio-Tobón et al., 2013)

#### 3.1.5 Ultrasound-assisted extraction

Ultrasound-assisted extraction (UAE) is a sustainable method for obtaining natural dyes using high-frequency sound waves. It breaks plant cell walls, enhancing pigment release. This technique requires less time, energy, and chemicals while improving yield and preserving heat-sensitive compounds. UAE is widely used in textiles, food, and cosmetics, making natural dye production more efficient and eco-friendly (Chemat et al., 2017).

#### 3.1.6 Solid-phase extraction

Solid-phase extraction is a technique that utilizes a solid adsorbent selected based on the properties of the target dye compounds. SPE is conceptually similar to liquid-liquid extraction, as both techniques involve the distribution of specific compounds between two phases. In SPE, various adsorbents, including silica-based, carbon-based, and clay-based resins, are commonly employed. The solid adsorbent in SPE enhances the concentration and purification of the target constituents. Compared to liquid-liquid extraction, SPE offers several advantages, including higher recovery rates, reduced formation of emulsions, and simpler operational procedures (Sobanska et al., 2018).

#### 3.1.7 Surfactant-mediated technique

It involve the extraction and concentration of hydrophobic components from aqueous solutions using nonionic surfactants. In this process, the target constituents are transferred from the aqueous phase into the surfactant-rich phase. These techniques are increasingly being combined with ultrasound or microwave irradiation to enhance efficiency. The primary advantage of surfactant-mediated extraction is its effectiveness in extracting constituents that exhibit strong interactions with the surfactant medium, thereby improving the overall extraction performance (Frankewich and Hinze, 1994; Ali, 2011)

Several industries have successfully integrated advanced natural dye extraction techniques. Stony Creek Colors (USA) produces plant-based indigo dye using microbial fermentation for denim. Colorifix (UK) uses engineered microorganisms for textile dyes. Givaudan (Switzerland) applies ultrasound extraction for cosmetic pigments, while Oterra (Denmark) produces food colorants through fermentation. These examples demonstrate how innovative methods are transforming natural dye production in textiles, cosmetics, and food industries

### 3.2 Innovative methods

Innovations in extraction technologies have improved yield and consistency. For instance, enzymatic extraction and grinding assisted microwave methods ensure a higher concentration of dye compounds while reducing resource consumption. Some innovative extraction method such as electrochemical extraction is also being examined for natural dye extraction. These techniques also make it easier to extract colors from previously underutilized sources, such as agricultural waste and invasive plants (Rahaman and Khan, 2024). While these advanced methods vary in equipment cost and scalability, they consistently outperform traditional techniques in terms of yield and environmental sustainability. Several companies have successfully implemented advanced biotechnological approaches for large-scale natural dye production. Stony Creek Colors (USA) utilizes fermentation-based extraction and purification of indigo dye from *Indigofera* species. Their patented technique (Bellos, 2019; Bach, L. and Cannon, D. (2018)) focuses on stabilization of bioindigo pigment through filtration and enzymatic treatment, allowing compatibility with industrial denim dyeing systems. The company supplies major denim brands like Levi Strauss & Co., demonstrating both scalability and market acceptance. Their vertically integrated model—from regenerative farming to dye extraction—highlights commercial viability while maintaining a sustainable supply chain. Colorifix (UK) employs a fully biological dyeing process, where microorganisms are genetically engineered to produce specific pigments. The workflow includes (i) DNA sequence identification for color genes from natural organisms, (ii) insertion of these genes into microbial hosts such as *E. coli*, and (iii) fermentation-based dye production. The dyeing process involves printing the microbes directly onto fabric where they fix the color, eliminating the need for hazardous mordants or salts. Colorifix’s process is protected under patents (Ajioka et al., 2017) and emphasizes water and chemical savings of up to 90%.

#### 3.2.1 Enzymatic extraction

Enzymatic extraction is a green technique that uses specific enzymes to degrade plant cell walls and release dye compounds more effectively. Commonly employed enzymes include cellulases, pectinases, and laccases. Cellulase and pectinase treatments have been reported to improve anthocyanin and flavonoid extraction yields by 20–30%, especially from berries, onion skins, and flower petals. These enzymes not only improve pigment release but also enhance color stability and reduce processing time. Such enzyme-assisted processes offer advantages over solvent extraction by being non-toxic, energy-efficient, and biodegradable, making them ideal for eco-friendly dye production (García-Cruz et al., 2022).

#### 3.2.2 Grinding assisted microwave extraction

Grinding-assisted microwave extraction is an advanced and innovative technique originally developed for the synthesis of organic and inorganic compounds (Yadav et al., 2024). Its adaptation for natural dye extraction is a recent advancement and has shown promising results in early-stage experimental studies. This method combines mechanical grinding with simultaneous microwave irradiation to enhance the extraction process. In this technique, the plant material, either with or without a solvent, is ground in a tubular glass mortar and pestle setup, which is integrated into a microwave oven equipped with a condenser. The mechanical grinding facilitates the breakdown of plant cell structures, while microwave irradiation promotes rapid heating and efficient transfer of the dye components into the solvent or extraction medium. Some experimental studies (Sowbhagya and Chitra, 2010) demonstrate that GAME able to improve extraction efficiency by 10–15%, reduces solvent use, and maintains better pigment integrity compared to conventional MAE. These findings support its superiority as a green and cost-effective alternative for plant-based dye extraction. Additionally, it offers a cost-effective solution while maintaining environmental sustainability.

#### 3.2.3 Economic feasibility and challenges

Although as given in **Table 1** modern method are efficient but high cost of advanced natural dye extraction methods poses challenges for small-scale producers due to expensive equipment and limited resources. However, technological advancements, government support, and shared facilities can reduce costs. Growing consumer demand for eco-friendly products also creates opportunities.

**Table 1:** Contrasts traditional and modern natural dye extraction methods in terms of efficiency, cost, scalability, and sustainability (Jiménez-Carmona et al., 1999; Tao et al., 2014; Manzocco et al., 2015; Sánchez-Camargo et al., 2019; Rather et al., 2024; Liu et al., 2023).”.

Parameters	Traditional Methods	Modern/Advanced Methods
Extraction Yield	Low or Moderate (25–40%)	High to Very High (60–98%)
Extraction Time	Long time (2–6 hrs)	Short (10–60 min)
Solvent/Energy Use	High	Comparable Low
Color Fastness	Moderate (3.0–3.5)	High (4.5–5.0)
Environmental Impact	High (more water and energy are required)	Low (minimal emissions, green solvents, less waste)
Equipment Cost	Low (general setups like boiling vessels)	High (advanced reactors, microwave systems, supercritical vessels)
Scalability	Moderate (labor-intensive, batch-based)	High (automated, efficient, continuous-flow compatible)
Sustainability	Limited due to high resource demand	Strong (eco-friendly inputs, supports circular economy)

As research improves efficiency, the cost of ultrasound and microbial is expected to drop, making sustainable dye production more accessible for artisans and small businesses. Scaling up natural dye production faces challenges like regulatory hurdles, environmental concerns, and economic barriers. Strict safety standards and certification costs limit small producers. Large-scale extraction may require high water and energy use, while waste management remains an issue. High investment costs and inconsistent dye yields also pose difficulties, requiring further research, policy support, and sustainable innovations. However, it was reported that *Escherichia coli* and *Pseudomonas putida* have been genetically modified to produce indigo from tryptophan via the expression of tryptophanase enzyme. Similarly, *Saccharomyces cerevisiae* has been engineered to biosynthesize anthocyanin (Xu et al., 2022; Chandel et al., 2024). These microbial systems allow pilot-scale fermentation of dyes, reducing dependency on agricultural land and ensuring batch-to-batch pigment consistency (Liu et al., 2023). Several lifecycle assessments (LCAs) and techno-economic analyses have been conducted to evaluate the environmental and operational trade-offs between modern and traditional natural dye extraction methods. It was reported that that Pressurized Liquid Extraction (PLE) and Microwave-Assisted Extraction (MAE) reduced overall energy consumption by up to 60% and solvent usage by 40–70% compared to traditional boiling, while maintaining superior dye quality and extraction efficiency. However, these methods require specialized equipment, which can increase initial capital costs by 3–5 times, depending on the scale of operation (Jiménez-Carmona et al., 1999). In terms of environmental impact, Supercritical Fluid Extraction while highly efficient demands significant energy to maintain high pressures (often above 35 MPa), although the use of recyclable CO_2_ as a solvent reduces chemical waste generation. Conversely, traditional boiling consumes large volumes of water (10–15 L per kg of plant material), generates considerable wastewater with plant residues, and offers limited dye recovery (~30–40%). Moreover, comparative LCA have shown that SFE systems produce 80% less solid waste and require 50% less post-extraction treatment than conventional methods (Tao et al., 2014). These assessments highlight that although modern methods may involve higher upfront costs and technical complexity, they offer long-term benefits in terms of sustainability, process efficiency, and waste reduction. As such, their adoption in commercial dye production is increasingly supported by regulatory incentives and green certification frameworks

## 4 Advantage of modern and innovative methods

The advantages are given followings

### 4.1 Enhanced extraction efficiency and yield

Advanced techniques significantly improve the efficiency and yield of dye extraction processes by optimizing parameters such as temperature, pressure, and solvent interactions, ensuring maximum recovery of dye components from plant materials. For example, biotechnological advancements like microbial fermentation have enabled the production of natural indigo from bacteria, reducing the need for chemically intensive processes. Companies like Stony Creek Colors in the U.S. use sustainable agriculture and fermentation techniques to produce high-purity plant-based indigo for the textile industry (Chandel et al., 2024). Genetically modified bacteria like as *Escherichia coli* and *Pseudomonas putida*, which have been engineered to synthesize indigo more efficiently. Researchers have modified these bacteria to express the tryptophanase enzyme, which converts tryptophan into indole, a precursor for indigo production (Chandel et al., 2024). Another promising innovation is engineered yeast strains like *Saccharomyces cerevisiae*, which are modified to produce flavonoid-based dyes such as anthocyanins. These dyes, commonly found in berries and flowers, can now be produced through fermentation, reducing agricultural land use and water consumption (Xu et al., 2022).

### 4.2 Improved dye quality and color vibrancy

These methods provide better control over extraction conditions, leading to consistent dye quality and enhanced color vibrancy, making the dyes suitable for a wide range of applications.

### 4.3 Reduced environmental impact

Advanced techniques often require lower energy input, minimal solvent usage, and generate less waste, thereby reducing the overall environmental footprint compared to conventional methods. Many researchers and industries are now exploring sustainable dye extraction from agricultural waste, food by-products, and invasive plant species. Theses researches reduce environmental impact and enhance resource efficiency. The use of food waste, such as onion peels, pomegranate rinds, avocado seeds, and black carrot residues, for dye production is now being practiced (Mohan et al., 2020). These contain rich natural pigments like flavonoids, tannins, and anthocyanins, which can be effectively used in textile dyeing. These sources help reduce food waste while providing cost-effective and eco-friendly dye alternative.

### 4.4 Sustainability and diversification of sources

By enabling the use of non-traditional and diverse raw materials, these techniques expand the range of natural dye sources, reducing reliance on conventional resources and promoting sustainable practices in dye production.

## 5 Conclusion

Modern and innovative methods are more superior to traditional natural dye extraction process. Innovative methods are superior because these techniques make feasible to extract dyes from unconventional and previously underutilized sources obtained from agricultural waste such as byproducts from crops, such as fruit peels, seeds, and stems, which were once discarded, are now valuable sources of natural dyes. Species that pose ecological challenges can be repurposed for dye extraction, turning an environmental problem into a resourceful solution. The intersection of tradition and modern technology is driving a renaissance in natural dye production. By leveraging innovative scientific methods, sustainable practices, and digital tools, the industry is overcoming historical limitations and paving the way for a vibrant future. These innovations not only preserve the cultural legacy of natural dyes but also contribute to a more sustainable and ethical global economy

Beyond textiles, modern natural dye technologies are influencing other industries such as fashion, eco-tourism, cosmetics, and home décor. Sustainable fashion brands are embracing natural dyeing techniques to reduce environmental impact, while eco-tourism initiatives promote traditional dye-making as a cultural and heritage experience. Additionally, interior design and cosmetic industries are increasingly adopting plant-based colorants for eco-conscious consumers.

By integrating modern extraction techniques and circular economy principles, natural dye production is transforming multiple industries, ensuring a more ethical, eco-friendly, and commercially viable future on a global scale.
